# Double-layered cell transfer technology for bone regeneration

**DOI:** 10.1038/srep33286

**Published:** 2016-09-14

**Authors:** Keiko Akazawa, Kengo Iwasaki, Mizuki Nagata, Naoki Yokoyama, Hirohito Ayame, Kazumasa Yamaki, Yuichi Tanaka, Izumi Honda, Chikako Morioka, Tsuyoshi Kimura, Motohiro Komaki, Akio Kishida, Yuichi Izumi, Ikuo Morita

**Affiliations:** 1 Periodontology, Department of Hard Tissue Engineering, Graduate School of Medical and Dental Sciences, Tokyo Medical and Dental University (TMDU), 1-5-45 Yushima, Bunkyo-ku, Tokyo, 113-8510, Japan; 2 Department of Nanomedicine (DNP), Graduate School of Medical and Dental Sciences, Tokyo Medical and Dental University (TMDU), 1-5-45 Yushima, Bunkyo-ku, Tokyo, 113-8510, Japan; 3 Life Science Laboratory, Research and Development Center, Dai Nippon Printing Co., Ltd., 1-1-1 Kaga-cho, Shinjuku-ku, Tokyo, 162-8001, Japan; 4 Department of Comprehensive Reproductive Medicine, Graduate School of Medical and Dental Science, Tokyo Medical and Dental University (TMDU), 1-5-45 Yushima, Bunkyo-ku, Tokyo, 113-8510, Japan; 5 Department of Pediatrics and Developmental Biology, Graduate School of Medical and Dental Science, Tokyo Medical and Dental University (TMDU), 1-5-45 Yushima, Bunkyo-ku, Tokyo, 113-8510, Japan; 6 Department of Material-based Medical Engineering, Institute of Biomaterials and Bioengineering, Tokyo Medical and Dental University (TMDU), 2-3-10, Kanda-Surugadai, Chiyoda-ku, Tokyo 101-0062, Japan; 7 Department of Cellular Physiological Chemistry, Graduate School of Medical and Dental Sciences, Tokyo Medical and Dental University (TMDU), 1-5-45 Yushima, Bunkyo-ku, Tokyo, 113-8510, Japan

## Abstract

For cell-based medicine, to mimic *in vivo* cellular localization, various tissue engineering approaches have been studied to obtain a desirable arrangement of cells on scaffold materials. We have developed a novel method of cell manipulation called “cell transfer technology”, enabling the transfer of cultured cells onto scaffold materials, and controlling cell topology. Here we show that using this technique, two different cell types can be transferred onto a scaffold surface as stable double layers or in patterned arrangements. Various combinations of adherent cells were transferred to a scaffold, amniotic membrane, in overlapping bilayers (double-layered cell transfer), and transferred cells showed stability upon deformations of the material including folding and trimming. Transplantation of mesenchymal stem cells from periodontal ligaments (PDLSC) and osteoblasts, using double-layered cell transfer significantly enhanced bone formation, when compared to single cell type transplantation. Our findings suggest that this double-layer cell transfer is useful to produce a cell transplantation material that can bear two cell layers. Moreover, the transplantation of an amniotic membrane with PDLSCs/osteoblasts by cell transfer technology has therapeutic potential for bone defects. We conclude that cell transfer technology provides a novel and unique cell transplantation method for bone regeneration.

Recent progress in tissue engineering has made it possible to regenerate tissues using *ex vivo*-expanded cells. A number of cell transplantation methods have been developed to regenerate tissues lost during disease progression^1^. For example, various studies have previously reported the use of several methods for the regeneration of hard tissues including topical injection of cells^2^, transplantation of cells cultured on/with scaffold^3^, and transplantation of cells in a sheet format^4^. The results of cell transplantation are closely related to the method of transplantation, and cell transplantation methods must be further optimized for each regenerative therapy; thus, new methods are still needed.

Appling the photolithographic technique used in printing and semiconductor manufacturing, we have developed a novel method of cell transplantation called “cell transfer technology”, which enables the transfer of cultured cells to the surface of the scaffold material^5^. This cell transfer technology utilizes the hydrophilic/hydrophobic nature of the transfer base surface, which is provided by the partial degradation of a tetraethylene glycol (TEG) layer by ultra violet irradiation. The adjustment of hydrophilicity/hydrophobicity of the transfer base controls the strength of cell adhesion and permits cell transfer from the base surface to the scaffold surface. One of the unique characteristics of this cell transfer technology is the extensive control of cell position upon cell transfer; it is possible to position cultured cells in such a way to form desired patterns on scaffold materials such as matrix gel and amniotic membrane, similar to printing letters and pictures on paper. We have reported an improvement in blood flow after transplantation of scaffold materials with endothelial cells, which mimic the topology of a capillary, using this cell transfer technology in mice^5^. In addition, it is also possible to fabricate sheet-like cell transplantation materials using this cell transfer technology, wherein the most important factor is cell number, not position. For the regeneration of hard tissues, we have previously reported the therapeutic potential of sheet-like cell transfer. We demonstrated bone regeneration after transplantation of osteoblasts and mesenchymal stem cells (MSC), from the periodontal ligament, into a calvarial bone defect mouse model, and a periodontal defect rat model, respectively^6^,^7^. However, it is still possible to improve on the methodology for hard tissue regeneration using this cell transfer technique, taking into account the specific cell types needed for regeneration and the mode of cell transplantation.

It is generally accepted that transplanted cells communicate with host cells and microenvironments through secreted factors, direct cell-cell, and cell-matrix contact. It is possible that these interactions affect the results of cell-based treatment by regulating growth, differentiation and survival of the transplanted cells. The capacity of a single and specific cell type to form defined tissues has long been used for cell-based therapies. However, some recent reports have demonstrated that the transplantation of multiple cell types increased regeneration through enhancing cell survival and cellular activity when compared to single cell type transplantation^8^,^9^,^10^. These results emphasized the advantages of combined cell transplantation using several cell types over single cell type.

We aimed to expand the range of applications of cell transfer technology in hard tissue regeneration, by investigating the transfer of two different types of cells. Moreover, we investigated if two-layer cell transplantation of MSC and osteoblasts, using this cell transfer technology, could enhance bone regeneration in a calvarial bone defect mouse model and examined the therapeutic potential of this method.

## Methods

### Cell culture

Human calvaria osteoblasts (HCO) (ScienCell, Carlsbad, CA, USA), normal human dermal fibroblasts (NHDF) (Lonza, Walkersville, MD, USA), and human umbilical vein endothelial cells (HUVEC) (Lonza) were purchased for use in this study. Mouse osteoblasts (KUSA-A1) were a gift from Professor Akihiro Umezawa of the National Institute for Child Health and Development. Cell lines were cultured with following media: Osteoblast Medium (ScienCell) (for HCO cell), Dulbecco’s Modified Eagle’s Medium (DMEM, Life Technologies, Carlsbad, CA, USA) with 10% fetal bovine serum (FBS) (for NHDF cells), endothelial cell growth medium (EGM2, Lonza) (for HUVEC cells) and αMinimum Essential Medium (αMEM, Life Technologies) with 10% FBS (for KUSA-A1 cells). Cell lines were cultured at 37 °C in 5% CO_2_, changing cell media every 3 days. Periodontal ligament stem cells (PDLSCs), which are putative tissue stem cells located in the tooth-supporting ligament tissue, possess MSC-like characteristics *in vitro*. We used this dental stem cell population as our study focused on the regeneration of tooth-supporting tissues. Periodontal ligament tissues were obtained from premolars extracted from healthy subjects. We collected extracted teeth from patients that visited the Tokyo Medical and Dental University Dental Hospital (patients signed a consent form) after obtaining written informed consent from all subjects. The protocol was approved by the clinical research ethics committee at the Tokyo Medical and Dental University (#723) and all experiments were performed in accordance with the Ethical Guidelines for Medical and Health Research Involving Human Subjects by the Japanese Ministry of Health, Labour and Welfare. Periodontal ligament tissues were minced using a surgical knife for 5 min in 500 μL of a digestion solution with the following composition: αMEM containing collagenase type I (3 mg/mL, WAKO Pure Chemicals, Osaka, Japan) and dispase (4 mg/mL, Life Technologies). The digestion solution (500 μL) was added to the minced periodontal ligament solution, for enzymatic digestion in a 37 °C water bath for 30–60 min under continuous agitation. After enzymatic digestion, the reaction solution was centrifuged and the supernatant was discarded. Cell growth medium (αMEM with 15% FBS, 10 mL) was added to obtain a single cell suspension. The cell suspension was passed through a cell strainer (pore size, 70 μm) (BD Falcon, Bedford, MA, USA) to remove debris, and the flow through was applied to a culture dish. The culture medium was changed every 2 to 3 days, and colony-forming PDLSCs were harvested and passaged. PDLSCs with a passage number of less than five were used for all experiments in this study.

### Labeling of cultured cells

Cultured cells were labeled using PKH26 or PKH67 (Sigma, St Louis, MO, USA) according to the manufacturer’s protocol. Green fluorescence protein (GFP)- expressing PDLSCs (PDLSC-GFP) and osteoblasts (KUSA-GFP) were established and cultured as previously described^6^,^7^.

### Preprocessing of the amniotic membrane

Human fetal membranes were harvested from patients undergoing cesarean delivery at the Tokyo Medical and Dental University Hospital. Written informed consent was obtained from all subjects. This research protocol was approved by the clinical research ethics committee at the Tokyo Medical and Dental University (TMDU). The amniotic membrane was washed, cut into small pieces, and incubated for 1 h at 37 °C in 0.02% ethylenediamine tetraacetic acid (EDTA, Dojindo, Tokyo, Japan) in phosphate-buffered saline (PBS). After incubation, the chorion was mechanically removed from the amnion using a cell scraper and tweezers. The resulting amnion was stored in DMEM containing 50% glycerol (WAKO) at −80 °C until use. Cell components of the amnion were removed by high hydrostatic pressure treatment as previously reported^11^,^12^.

### Cell transfer onto amnion

**Figure 1:**
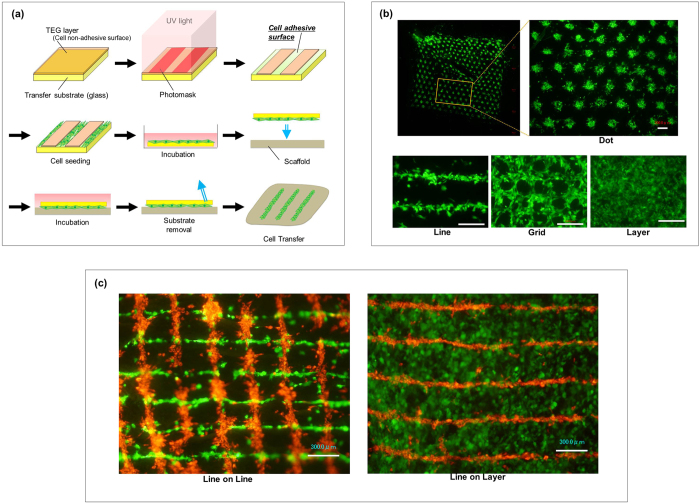
Cell transfer technology and double cell transfer. (**a**) Schematic diagram of cell transfer technology. We coated the surface of glass substrate with tetraethyleneglycol (TEG, brown) and the layer was partially degraded by UV irradiation to prepare hydrophilic cell adhesive surface (green). The TEG area covered with photomask remained as non-cell adhesive surface. Cells to be transferred were poured onto the substrate and incubated to allow the cells to adhere to the substrate surface. Transfer substrate with cells was then placed onto the scaffold (amnion) in the direction of cell surface down. Cells were further cultured and transfer substrate was carefully removed subsequently. Cells are transferred onto scaffold surface. (**b**) Various cell patterning by using cell transfer technology. PDLSC (GFP; green) were transferred onto amnion using patterning base for dot, line, grid and layer placement. Boxed area was closed-up on the right figure. Bar = 300 μm (**c**) Double cell transfer using line patterning and layer transfer base. Fibroblasts (PKH26; red) were transferred using line patterning base and PDLSC (GFP; green) were transferred overlaying the fibroblasts displacing 90 degrees (left). PDLSC in layer structure was transferred on fibroblasts in line pattern (right). Bar = 300 μm.

Cell transfer onto the amnion was performed following previously described methods that are summarized in Fig. 1a 5,6,7,13. Briefly, TEG-coated glass substrate was treated with UV irradiation and the transfer substrate was prepared. Cultured cells (5×10^5^) were seeded onto the transfer substrate (1 cm × 1 cm) and incubated in 5% CO_2_ for 3 h at 37 °C. Following the incubation period, the transfer base was placed downward onto the amnion. Growth medium was added and cultured. After 5–18 h incubation, the transfer substrate was gently removed using tweezers. Cells that were transferred to the amniotic membrane were examined under a fluorescence microscope (Keyence BZ800, Keyence, Osaka, Japan) and bright field microscope. In observing transferred cells *in vitro*, the scaffold (amniotic membrane) was not visualized in figures.

### Transplantation of cell-transferred amniotic membranes in calvarial bone defects

All animal study protocols and procedures were approved by the Animal Care Ethics Committee of the Tokyo Medical and Dental University. All experiments were carried out in accordance with the approved guidelines by Science Council of Japan for proper conduct of animal experiments. We used 16 male nude mice (BALB/c Slc-*nu*/*nu*, Sankyo Labo Service Corporation, Tokyo, Japan) that were 8 weeks old for this experiment. Isoflurane inhalation (Abbott Laboratories, Queenborough, UK) was used to anesthetize mice. After making an incision in the skull skin, the periosteum was carefully removed and two critical sized bone defects were created on the calvaria (3.75 mm diameter) using a trephine bur (ACE Dental Implant System, Brockton, MA, USA). The cell-transferred amniotic membrane was then trimmed and transplanted to cover the bone defect and the wound was closed using a 7–0 silk suture (Mani, Tochigi, Japan).

### Micro-computed tomography, imaging, and measurement of bone regeneration in calvarial defects

Calvarial bone defects were examined by micro-computed tomography (micro-CT) (inspeXio SMX100CT, Shimadzu Co., Kyoto, Japan) and three-dimensional images were constructed from the scan data using a VG Studio MA2.0 (Volume Graphics, Heidelberg, Germany). Bone regeneration was measured and quantified using image analysis software (BZ- analyzer, Keyence, Osaka, Japan).

### Histological analysis

To obtain cross sectional images of the cell transferred-amnion, frozen sections were created using freezing medium (Tissue-tek, Sakura Finetek Japan, Tokyo, Japan) after fixing the amnion with 4% paraformaldehyde in PBS. For the histological analysis of mouse calvaria, mice were sacrificed and the skull block was isolated and fixed with 4% paraformaldehyde in PBS. Calvaria bone was demineralized in 10% EDTA for 4 weeks at 4 °C. Paraffin embedded sections were made for histological observations.

### Statistical analysis

All results are expressed as mean ± standard deviation (S.D). Statistical differences between experimental groups were examined using JMP version 5.0 (SAS Institute Inc, Cary, NC, USA). For the comparison of experimental groups, analysis of variance (ANOVA) was first used. When the significant variation was found by ANOVA, post-hoc analysis was performed using the Tukey’s test. Differences with p < 0.05 were considered significant.

## Results

### Transfer of double-layered cells onto the scaffold

This cell transfer technology allowed us to control the position of cells to the desired pattern on a micrometer scale (Fig. 1a). Figure 1b shows PDLSC position and transfer to the amniotic membrane in dot, line, and grid patterns. With this transfer technology, it is also possible to transfer cells as a layer using a transfer base with a whole cell adhesive surface (Fig. 1b). To examine the potential of multiple cell type transfer using by this method, we repeated cell transfer onto the same amnion surface area using a patterned-cell transfer base (double-cell transfer). First we transferred fibroblasts using a line pattern transfer base and transferred PDLSCs onto it using a line pattern base displaced by 90 degrees. Two cell types, fibroblasts and PDLSCs, were placed in a grid-like pattern onto the amnion surface (Fig. 1c). Fibroblasts and PDLSCs were also transferred in line and layer structures, respectively (Fig. 1c).

**Figure 2:**
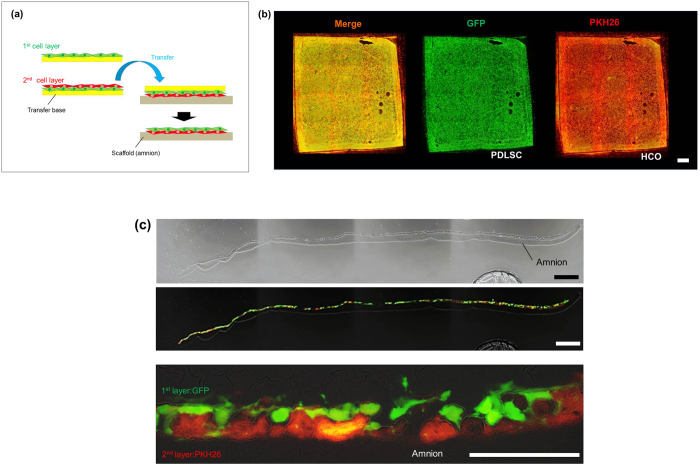
Double-layered cell transfer onto amnion. (**a**) Schematic diagrams of the double-layered transfer. Cells of the first layer (green) were seeded on the transfer substrate and cultured for 3 hours. Then, the cells of the second layer (red) were seeded onto the cells of the first layer. After 30 minutes to 3 hours incubation, transfer substrate bearing two layers of cells was placed onto the amnion to make direct contact between cells and scaffold surface. We removed transfer substrate from amniotic membrane after 5 hours. (**b**) Fluorescence microscope image of the double-layered cell transferred-amnion. Two types of cells, PDLSC-GFP (green) and HCO-PKH26 (red), were observed as a cell layer on amnion. (**c**) A cross-sectional image of the double-layered cell transferred-amnion. PDLSC (GFP, green) and HCO (PKH26, red) maintained double-layer structures on amnion. Upper: Phase-contrast microscopic image at lower magnification, bar = 300 μm. Middle: Fluorescence microscope image at lower magnification, bar = 300 μm. Bottom: higher magnification image, bar = 100 μm.

Double-cell transfer was successful when using a patterned transfer base. However, it failed when both cell types were transferred as layers (see Supplementary Figure 1). We observed cell detachment at the center of the cell layers, especially in the second layer. Based on these results, we tried to transfer two cell types using a single cell transfer process, which consisted of making the double cell layer on the transfer base in advance. Figure 2a depicts a schematic diagram of this method (double-layered cell transfer). First, we seeded and cultured the first layer cells onto the transfer base. Next, the second layer cells were poured on the first layer and cultured, allowing the second cell layer to adhere to the first. Then, we placed the transfer base, which contained double-layered cells, to produce direct contact between the cells and the scaffold. After incubation, double-layered cells were successfully transferred onto the amnion. Figure 2b shows a fluorescent microscopic view of the amnion using GFP-labeled PDLSCs (PDLSC-GFP, green) and PKH26-labelled human osteoblasts (HCO-PKH26, red), following double-layered cell transfer. After cell transfer, both PDLSCs and HCOs showed sheet-like structures. Further, to confirm cell transfer, we examined double-layered cell transfer in cross sections. As demonstrated in Fig. 2c, overlapping cell layers were observed on the amnion, implying that a successful cell transfer was obtained with this method.

When two cell types were mixed before seeding onto the transfer base, and then transferred through a single transfer, we observed the formation of cell aggregates and unbalanced cell density of the transferred cells (see Supplementary Figure 2). From these results, it was revealed that the double-layered cell transfer was the best method to transfer two cell types in an overlapped cell layer structure. We also obtained a successful triple-layered cell transfer using this method (see Supplementary Figure 3).

### Double-layered cell transfer using various cell type combinations

**Figure 3:**
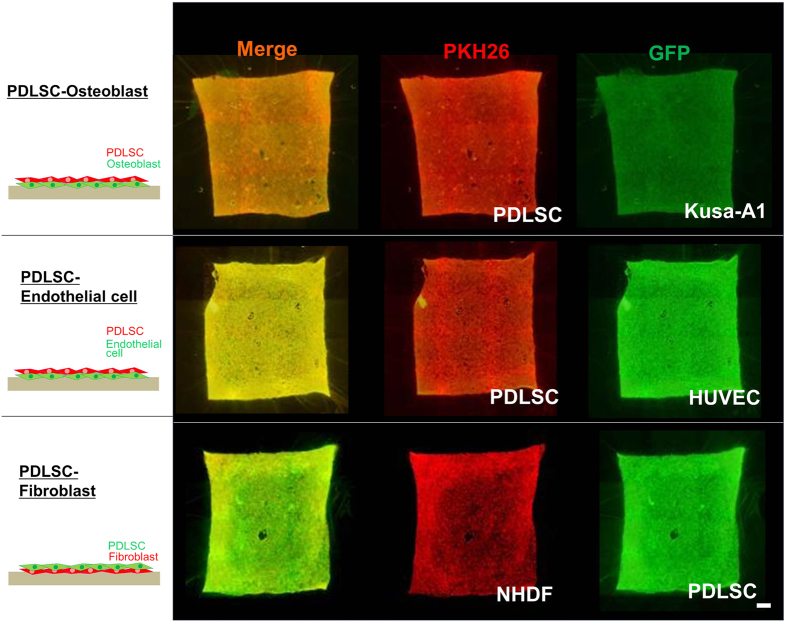
Double-layered cell transfer using various cell types. Application of double-layered cell transfer to various cell types. Double-layered cell transfer was investigated using the combination of two cell types indicated. Successful cell transfer was observed in all cell type combinations demonstrated. Schema of double–layered cell transfer was shown in each cell type combinations (left). GFP (green) and PKH26 (red) were utilized for cell labelling.

We examined if double-layered cell transfer was applicable to cell type combinations other than PDLSCs and osteoblasts. We selected mouse osteoblasts (KUSA-A1), human endothelial cells (HUVEC), and human fibroblasts (NHDF) as the different cell types for the second layer. Figure 3 demonstrates the results of the double-layered cell transfer of KUSA-A1s, HUVECs, and NHDFs in combination with PDLSCs. Successful cell transfer was obtained with all tested cell type combinations. These results suggest that double-layered cell transfer could be applied to a wide variety of adherent cells.

### Stability of transferred cells after deformation of the amnion

**Figure 4:**
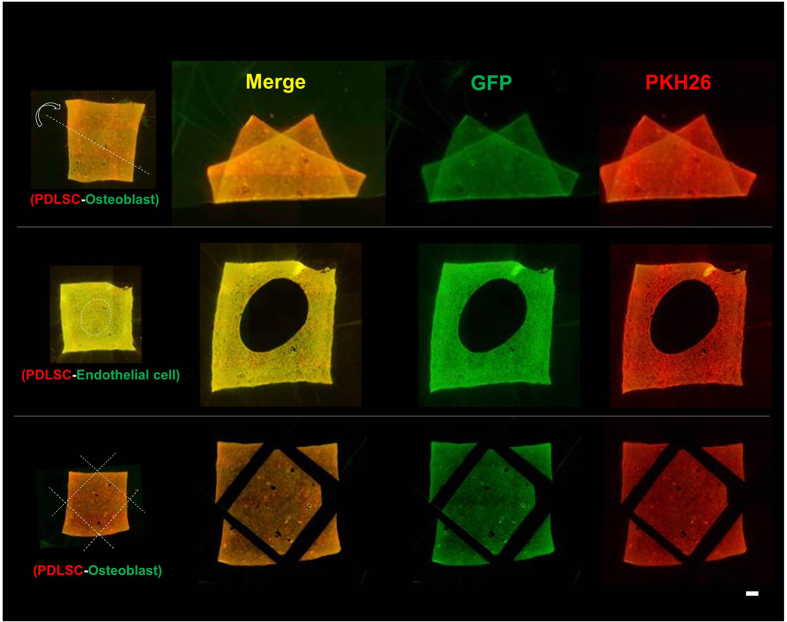
Stability of transferred cells upon trimming and deformation of amnion. Fluorescence microscopic images of amnion holding double-layered cells after deformation (top), holing (middle) and trimming (bottom) of the membrane. After double-layered cell transfer, using PDLSC (GFP, green) and KUSA-A1 (PKH26, red), amnion was folded along the dotted line (top). Circular hole was made in the middle of cell-transferred amnion (middle). We used PDLSC (GFP, green) and HUVEC (PKH26, red) for double cell transfer. Four corners were trimmed along by the dotted line after double-layered cell transfer (bottom). Despite of deformations and trimming of cell transferred amnion, cells were stably adhered onto the scaffold material. We used PDLSC (GFP, green) and KUSA-A1 (PKH26, red) for double cell transfer, except for holing. PDLSC was used for the first layer in all cell transfer tested.

A unique characteristic of this cell transfer technology is the stability of transferred cells on the scaffold material. We have previously reported the stability of transferred-cells on the amnion, despite wide mobility and deformations of the membrane, including stretching, wrinkling and folding^7^. We investigated the stability of transferred cells, using double-layered transfer, upon the deformation of the membrane. As demonstrated in Fig. 4, the two cell layers, transferred using this cell transfer technology, stably adhered to the scaffold material, despite folding, holing, and trimming of the membrane. Transferred cells did not detach from the membrane and maintained their layer structure after deformations such as stretching and wrinkling (Supplementary Video).

### Transplantation of PDLSC and osteoblast-transferred amnion in calvarial bone defects in mice

**Figure 5:**
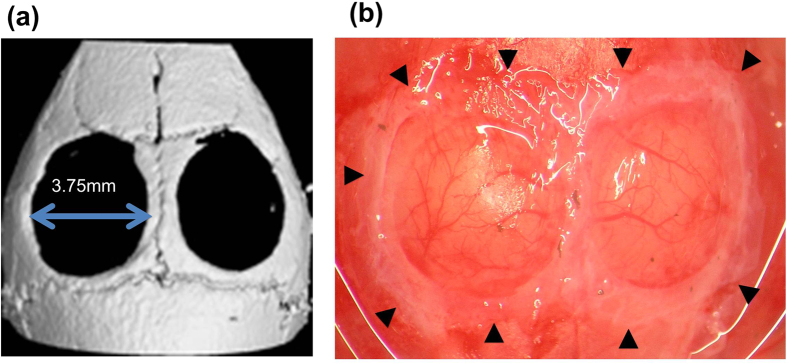
Transplantation of PDLSC and/or osteoblast-transferred amnion in calvaria bone defects. (**a**) Micro-CT image of mouse calvaria, immediately after the bone defect formation. We created two bone defects of diameter 3.75 mm. (**b**) Transplantation of cell-transferred amnion. Cell-transferred amnion was trimmed and placed to cover the calvaria bone defects. Arrow heads indicate the transplanted amnion.

**Figure 6:**
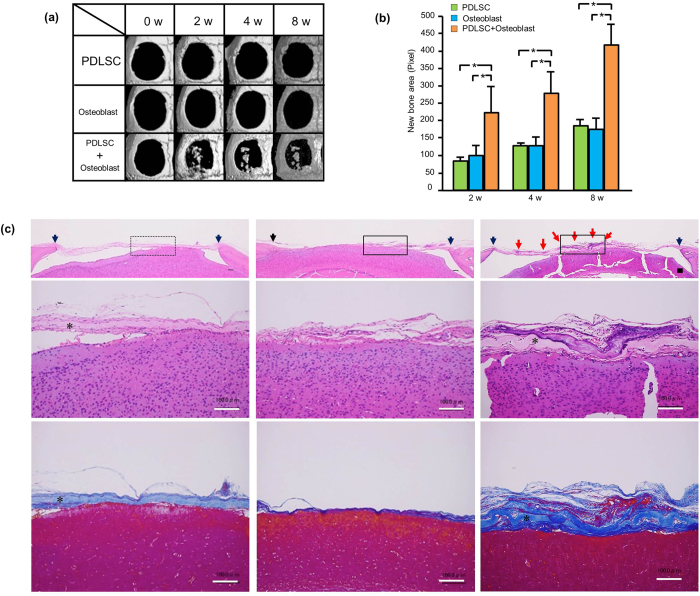
Bone regeneration by cell-transferred amnion. (**a**) Representative micro CT images of bone defects after the transplantation of cell-transferred amnion. Bone defects were treated with cell-transferred amnion (PDLSC, HCO or PDLSC + HCO) and CT images were taken periodically. In the single cell transplantation (PDLSC or HCO), bone healing was limited, while new bone-like tissue formation was observed in bone defects transplanted with double-layered cell-transferred amnion (PDLSC + HCO). (**b**) Quantification of new bone formation area on micro CT images. We constructed the three-dimensional image from scan data and the area of newly formed bone-like tissue was quantified using image analysis software on the images. Bone defects with PDLSC + HCO showed more bone-like tissue formation than with PDLSC or HCO alone. (*P < 0.05, Tukey-test) (**c**) Histological view of bone defects 8 weeks after cell transplantation. Frontal sections of bone defects from PDLSC (left), HCO (middle) and PDLSC + HCO (right) are shown. Pictures were taken after hematoxylin and eosin (upper and middle) or azan staining (bottom) at lower (upper) and higher (middle and bottom) magnification. Box frames in upper pictures indicate maginified field. Clusters of mineralized tissue were prominent in PDLSC + HCO group, compared with PDLSC or HCO group. Red arrow: mineralized tissue Black arrow heads: margine of bone defect. *Amniotic membrane. Bar = 100 μm.

To explore the possible applications of double-layered cell transfer for regenerative medicine, amnion with single- or double-layered cells were created using PDLSC or osteoblasts (HCO), and then transplanted to calvarial bone defects in mice. We have previously reported that the calvarial bone defects were completely filled by the transplantation of KUSA-A1-transferred amnion by 4 weeks^6^. To examine the effect of double-layer cell transfer in bone regeneration, we used HCOs, which possess diminished bone forming ability compared with KUSA-A1. Figure 5a,b show the micro-CT images of bone defects created in mice calvaria, and the amnion placed on the defects, respectively. Taking advantage of the cell stability after trimming the membrane, the cell-transferred amnion was trimmed in a circular shape to cover the bone defects (Fig. 5b). Figure 6a shows micro-CT images of bone defects after PDLSC, HCO, or PDLSC + HCO transplantation using this transfer technology. Limited bone healing was observed in bone defects transplanted with a single cell type-transfer amnion (PDLSC or HCO). In contrast, in bone defects that were transplanted with PDLSC + HCO, bone tissue formation was observed in both the peripheral area and the inside of the bone defect as island-like mineralized tissues. Quantification of bone-like tissue formation by micro-CT imaging revealed that transplantation of PDLSC + HCO significantly enhanced new bone formation, when compared to those of PDLSC or HCO cell transplantation (Fig. 6b), suggesting enhanced therapeutic potential of a double-layered cell transfer for bone regeneration. We confirmed new tissue formation histologically. As shown in Fig. 6c, bone defects were covered with thin layers of connective tissue in PDLSC- or HCO-transplanted bone defects, whereas PDLSC + HCO transplanted bone defects contained clusters of mineralized tissue, as seen through hematoxylin and eosin staining. Mineralized tissue clusters were also prominent in azan staining of sections from PDLSC + HCO transplanted bone defects confirming the enhanced capacity of PDLSC + HCO transplantation to form mineralized tissue, when compared to that of single transplantation of PDLSCs or HCOs.

## Discussion

The purpose of this study was to examine the feasibility of cell transfer technology involving two different cell types. We concluded that to perform double cell transfer, repetition of cell transfer procedures was required. This procedure facilitated the transfer of two different cell types when a patterned transfer base was used. Moreover, when a transfer base for cell layers was used, double-layered cell transfer was successful for the transfer of two cell types. We initially tried to transfer two cell types by repeating a single cell transfer. However, cells in the first and second layer detached from the amnion surface during the cell transfer procedure, especially at the center of the cell layer. This failed cell transfer was potentially due to differences in the attachment strength of the transfer base and the cell, and between the first and second cell layers. To avoid repeated cell transfer procedures, we produced double cell layers on the transfer substrate before the cell transfer procedure; this resulted in successful cell transfer. From these results, the possibility to control a wide range of arrangements of different types of cells on scaffold materials using cell transfer technology is suggested.

*In vivo*, unique topology often exists between functionally related cells; for example, this occurs between mesenchymal cells and the osteoblast layer in the periosteum, and between the cementoblast layer, which aligns on the tooth root surface, and the adjacent periodontal ligament fibroblasts. Therefore, it is possible that a double-cell transfer method is useful for cell transplantation procedures by mimicking the *in vivo* structure of two cell types. It is still unclear if our method could affect the polarity of transferred cells. This is an important point of investigation for the future studies. It has also been reported that co-transplantation of endothelium/endothelial progenitor cells in combination with stem cells could improve heart function after a myocardial infraction and the clinical status of limb ischemia, mainly through early vascularization^14^,^15^,^16^. The double-cell transfer method may be useful for this type of co-transplantation purpose, as we have successfully performed a double-layer cell transfer using endothelial cells.

We observed that the transferred cells stably adhered to the amnion despite folding and cutting of the material. This unique characteristic makes it possible to trim the cell-transferred amnion, thereby adjusting it to the size of the transplantation site, and to manipulate the cell-transferred material reliably through surgical procedures. In utilizing this unique feature of the cell-transferred amnion, we trimmed and adjusted the position of the material to fit circular bone defects in mouse calvaria with minimal disturbance to transferred cells upon transplantation. Moreover, because of the flexibility of the amnion, it is considered that this method is suitable wherein close contact between the cell layers and the transplantation site is required. In contrast, because of this flexibility, our construct lacks the space making capacity. It is considered that porous scaffold materials are needed in combination with our construct where space making is insufficient. Recently, the use of multiphasic scaffolds was introduced as a novel scaffold-based regenerative approach for periodontal tissues^17^,^18^,^19^,^20^. This scaffold was comprised of each element of periodontal tissues to mimic biomechanical characterization, and aimed to enhance periodontal wound healing. It is possible to apply double-layered cell transfer to this novel method by making cementoblast and periodontal ligament cell layers, employing the controllable cell topology and physical stability of transferred cells in our method; however, further study is needed for this application.

Transplantation of the amnion with both PDLSCs and HCOs resulted in more new bone formation than transplantation with PDLSCs or HCOs alone. This result suggests that PDLSC + HCO transplantation was effective in bone regeneration and that this double-layered cell transfer technology is applicable to regenerative medicine. In this study, we could not clarify the mechanisms of enhanced bone formation. Because cell transfer with a mixture of PDLSCs and HCOs failed, (Supplementary Figure 2), we could not compare bone formation between the PDLSC/HCO mixture and PDLSC/HCO double cell transfer. Thus, it is unclear if increased bone formation was caused by the double layer structure made using this technique. Enhanced bone formation could be derived from the direct differentiation of transplanted PDLSCs into osteoblasts, since PDLSCs have been shown to have osteoblastic differentiation capacity^21^,^22^. Moreover, some studies have suggested that MSCs enhance the survival and engraftment of co-transplanted cells. Masuda *et al*. reported that hematopoietic stem cells displayed better engraftment when transplanted with MSCs in bone marrow^23^. Sordi *et al*. also demonstrated that co-transplantation of splenic islets with MSCs enhanced the survival and engraftment of islets, and resulted in improved blood glucose levels in a diabetes mouse model^24^. Further studies are needed to elucidate the underlying mechanisms of enhanced bone formation by PDLSC + HCO transplants.

The volume of newly formed mineralized tissues in this study was not prominent, compared with those shown in previously published literature, such as the effect shown by a potent inducer of bone formation, BMP-2^25^. Sawyer *et al*. have demonstrated that 40–60% of the calvarial bone defect was filled with new mineralized tissue 4 weeks after BMP-2 application^25^. The volume of new mineralized tissue that we demonstrated using PDLSC/HCO-transferred amnion was smaller than that shown in Sawyer’s results. This limited bone healing is derived from the lower bone-forming activity of the transplanted cells (HCO) in this study. We have previously demonstrated the 100% bone defect filling at 4 weeks by transplanting the osteoblast cell line KUSA-A1, which possesses higher bone-forming activity, using cell transfer technology^6^. Thus, it is possible that the combination of PDLSC and HCO was not ideal for the bone defect healing used in this study. The identification of best combination of cells for bone formation will be the topic of the future research.

Previously, numerous methodologies of cell transplantation for bone regeneration have been developed and studied including direct cell injection^26^,^27^ and the use of ceramic scaffolds^28^,^29^, hydrogels (natural or synthetic polymer)^30^,^31^,^32^, and cell sheets^4^,^33^. Each method has unique characteristics such as biocompatibility, intrinsic bioactivity through cell-material interaction and easy surgical application. However, the best method has not yet been identified. Compared with the aforementioned methods, there are three unique characteristics in our method. First, our method enables positioning of cells in the desired pattern and the transfer of two different cell types to the scaffold surface, keeping the cell positioning. Two types of cells are mixed and randomly placed when using hydrogels and the control of the cell position is difficult^34^,^35^. InVERT molding method, utilizes a unique grid to place the cultured cells, making it possible to form unique three-dimensional structures in hydrogels^9^. However, the cellular positioning achieved by this method was quite different from that in our method and it is difficult to make three-dimensional structures that mimic bone-periosteum or cementoblast-periodontal ligament fibroblast layers with this method. Second, we can fabricate the cell transplantation material through simple steps with double or triple cell layers in a relatively short period of time. Our method could transfer two cell layers in 5.5 hours at minimum. Temperature-responsive culture dish is a widely studied method for cell sheet formation and several days of culture are required for cells to form a cell sheet^36^. Third, cells transferred by our cell transfer method could stably adhere to the membrane and hardly detached upon deformation and trimming of the membrane, which allows easy surgical application. The cell sheet, made with a temperature-responsive dish, shows some difficulties in cell sheet collection under high cell density or thick cell layer conditions, and it is too fragile to manipulate the sheet with surgical instruments and needs support materials for its positioning^37^. To overcome the weakness of cell sheet methods, Zhou *et al*. have reported the unique technology that the combined stromal cell-sheet techniques and biodegradable scaffolds^38^. Our method has three unique characteristics that enable the fabrication and transplant of unique cell transplantation materials, which cannot be achieved by other methodologies. The cell transplantation we demonstrated in Figs 5 and 6 fully utilized the aforementioned characteristics of our method including double cell layer transfer, trimming of the cell-bearing materials to the recipient site, and precise placement of the material, proving the clinical feasibility of the method.

Cell transplantation in cell sheet format is one of the promising methods for bone regeneration. It has been demonstrated that transplantation of MSC sheet either in combination with or without 3D scaffold materials induced bone regeneration^33^,^38^,^39^. MSC cell sheets for bone regeneration have been fabricated using several methods including temperature-responsive culture dish and cell scraper. In the particular case of osteogenic cell, cell scraper method will be easier and have wide application compared with temperature-responsive culture dish method. Our cell transfer technology has some advantages such as stable holding of cells on the sheet, easy manipulation of cell constructs and shorter culture period. Moreover, our method can bear multiple cell layers as have shown in the present study. On the other hand, our cell transfer method is applicable to a relatively small size bone defect. Recently, Berner *et al*. reported that delayed injection of fragmented cell sheet could regenerate large bone defects^40^. Suitable method selection is required depending on the various conditions of cell transplantation.

In the present study, we demonstrated that this cell transfer technology is effective for fabricating an amnion that holds two different cell layers. Our results suggest that this double-layered cell transfer could be a new cell transplantation method for cell-based regenerative medicine.

## Additional Information

**How to cite this article**: Akazawa, K. *et al*. Double-layered cell transfer technology for bone regeneration. *Sci. Rep.* **6**, 33286; doi: 10.1038/srep33286 (2016).

## Supplementary Material

Supplementary Information

Supplementary Video
