# Supplementary material for: Double-layered cell transfer technology for bone regeneration
Source: Sci Rep. 2016 Sep 14;6:33286. doi: 10.1038/srep33286 (PMC5021950; doi:10.1038/srep33286)
Supplement: Supplementary Information [file srep33286-s1.pdf]

Double-layered cell transfer technology for bone regeneration

Keiko Akazawa[1], Kengo Iwasaki[2], Mizuki Nagata[1], Naoki Yokoyama[3], Hirohito Ayame[3], Kazumasa Yamaki[3], Yuichi Tanaka[3], Izumi Honda[4], Chikako Morioka[5], Tsuyoshi Kimura[4], Motohiro Komaki[2], Akio Kishida[6], Yuichi Izumi[1] & Ikuo Morita[7]

[1]Periodontology, Department of Hard Tissue Engineering, Graduate School of Medical and Dental Sciences, Tokyo Medical and Dental University, 1-5-45 Yushima, Bunkyo-ku, Tokyo, 113-8510, Japan.

[2]Department of Nanomedicine (DNP), Graduate School of Medical and Dental Sciences, Tokyo Medical and Dental University, 1-5-45 Yushima, Bunkyo-ku, Tokyo, 113-8510, Japan.

[3]Life Science Laboratory, Research and Development Center, Dai Nippon Printing Co., Ltd., 1-1-1 Kaga-cho, Shinjuku-ku, Tokyo, 162-8001, Japan.

[4]Department of Comprehensive Reproductive Medicine, Graduate School of Medical and Dental Science, Tokyo Medical and Dental University, 1-5-45 Yushima, Bunkyo-ku, Tokyo, 113-8510, Japan.

[5]Department of Pediatrics and Developmental Biology, Graduate School of Medical and Dental Science, Tokyo Medical and Dental University, 1-5-45 Yushima, Bunkyo-ku, Tokyo, 113-8510, Japan.

[6]Department of Material-based Medical Engineering, Institute of Biomaterials and Bioengineering, Tokyo Medical and Dental University, 2-3-10, Kanda-Surugadai, Chiyoda-ku, Tokyo 101-0062, Japan.

[7]Department of Cellular Physiological Chemistry, Graduate School of Medical and Dental Sciences, Tokyo Medical and Dental University, 1-5-45 Yushima, Bunkyo-ku, Tokyo, 113-8510, Japan.

Supplementary information

1. Supplementary Methods
2. Supplementary Figures
3. Video Legends

1. Supplementary Methods

Double cell transfer

HCO was seeded onto transfer substrate (5×10^5) for 15 min and placed the transfer substrate onto amnion. After 3 hours incubation, we removed the transfer substrate used for HCO transfer and put another transfer substrate with PDLSC (5×10^5) on amnion where HCO was transferred. Twenty hours later, second transfer substrate was carefully removed from amnion. We observed cell transfer under fluorescence microscope.

Mixed cell transfer

2.5×10^5 of osteoblasts (HCO, PKH26-labelled) and PDLSC-GFP were mixed prior to seeding on transfer base. Total 5×10^5 cells of HCO and PDLSC were seeded on transfer base and cultured for 5 hours. Transfer base were placed on amnion and incubated for 8 hours. After the incubation, the transfer base was removed.

Triple-layered cell transfer

PDLSC (PKH26-labelled, 5×10^5) were cultured on transfer base for 1.5 hours. Cell suspension of HUVEC-GFP (5×10^5) were added onto PDLSC layer on transfer base and incubated another 1.5 hours. Then, third layer cells, NHDF (stained with Hoechst 33342, 5×10^5), were overlaid on two layers of cells. After another 1.5 hours incubation, transfer substrate was placed onto amnion. Five hours later, transfer base was removed to observe using fluorescence microscope.

2. Supplementary Figures

Supplementary Figure 1

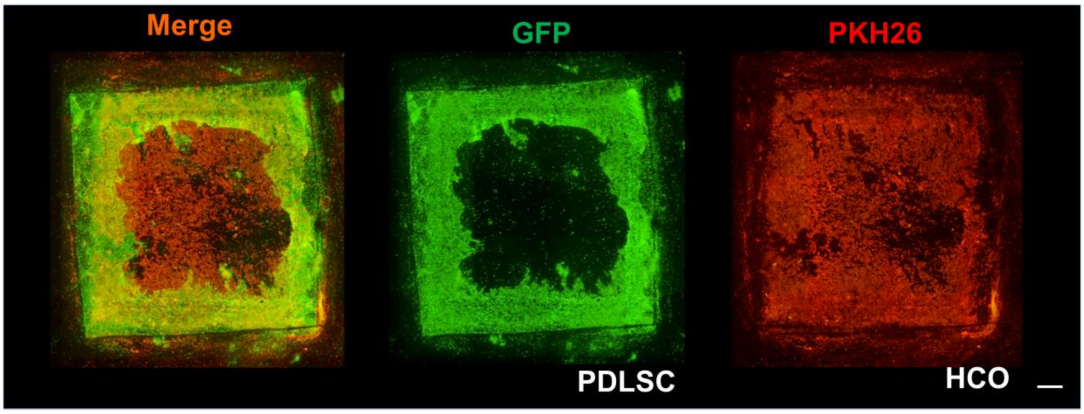

Double cell transfer by repeated single cell transfer. Fluorescence microscopic view of transferred cells. Osteoblasts (HCO, PKH26, red) were transferred onto amnion first and PDLSC (GFP, green) were transferred on HCO. Detachment of cells in center of PDLSC layer was significant. Some HCO came off from the amnion surface. Double cell transfer was not succeeded with this method. Bar=1 mm

Supplementary Figure 2

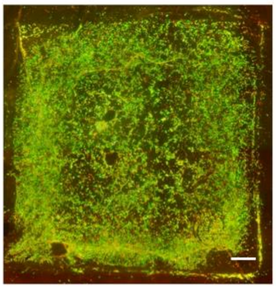

Fluorescence microscopic view of transferred cells after mixed cell transfer of osteoblasts and PDLSC. Osteoblasts (HCO, PKH26, red, $2.5*10^5$) and PDLSC (GFP; green, $2.5*10^5$) were mixed prior to seeding on transfer base and transferred onto amnion. Cell aggregation and empty space in cell layer was observed. Cells were not transferred evenly. Bar=1 mm.

Supplementary Figure 3

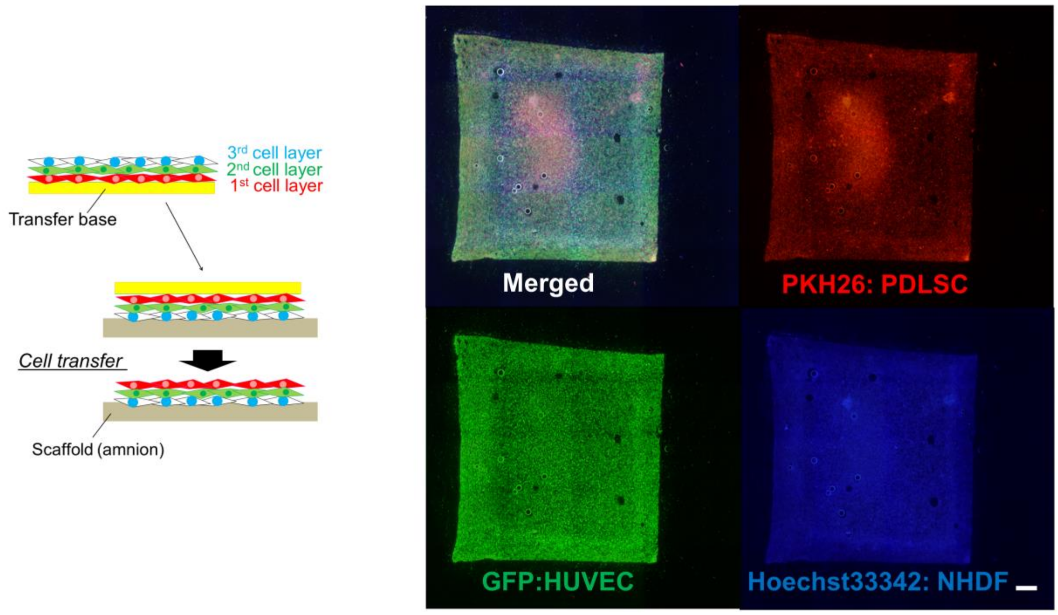

Triple-layered cell transfer by using cell transfer technology. Diagrams of triple-layered cell transfer (left). Three types of cells were cultured on transfer base in turn, 1.5 hour intervals and transferred onto amnion. Fluorescence microscopic view of transferred cells after triple-layered cell transfer (right). Three cell types were transferred onto amnion including PDLSC (1st layer, PKH26, red), HUVEC (2nd layer, GFP, green) and NHDF (3rd layer, Hoechst33342, blue). Bar=1 mm

3. Video Legends

Physical stability of cell transferred on amnion. By using double cell layer transfer, PDLSC (1st layer, GFP, green) and NHDF (2nd layer, PKH26, red) were transferred on amnion. Cells on amnion were observed under fluorescence microscope. The cell transferred-amnion were stretched and dragged strongly with surgical tweezers. Cells were stably retained on the membrane.
